# Impact of Preoperative Malnutrition on Patients with Pancreatic Neoplasms Post-Duodenopancreatectomy: A Retrospective Cohort Study

**DOI:** 10.3390/nu16121839

**Published:** 2024-06-12

**Authors:** Alvarez Pellegrinelli, Stefano Mancin, Alberto Brolese, Stefano Marcucci, Ornella Roat, Emanuela Morenghi, Sara Morales Palomares, Daniela Cattani, Diego Lopane, Alessandra Dacomi, Chiara Coldani, Giuseppina Tomaiuolo, Susy Dal Bello, Giovanni Capretti, Beatrice Mazzoleni

**Affiliations:** 1 Santa Chiara Hospital, 38122 Trento, Italy; alvarez.pellegrinelli@apss.tn.it (A.P.); alberto.brolese@apss.tn.it (A.B.); stefano.marcucci@apss.tn.it (S.M.); ornella.roat@apss.tn.it (O.R.); 2 Department of Biomedical Sciences, Humanitas University, 20072 Pieve Emanuele, Milan, Italy; emanuela.morenghi@humanitas.it (E.M.); giovanni.capretti@hunimed.eu (G.C.); beatrice.mazzoleni@hunimed.eu (B.M.); 3 IRCCS Humanitas Research Hospital, Rozzano, 20090 Milan, Italy; daniela.cattani@humanitas.it (D.C.); diego.lopane@hunimed.eu (D.L.); alessandra.dacomi@humanitas.it (A.D.); chiara.coldani@humanitas.it (C.C.); giuseppina.tomaiuolo@humanitas.it (G.T.); 4 Department of Pharmacy, Health and Nutritional Sciences (DFSSN), University of Calabria, 87036 Rende, Italy; sara.morales@unical.it; 5 Veneto Institute of Oncology IOV—IRCCS, 35128 Padua, Italy; susy.dalbello@iov.veneto.it

**Keywords:** malnutrition, pancreaticoduodenectomy, nursing, length of stay, nutritional assessment, surgery

## Abstract

Background: Preoperative malnutrition is a significant factor in patients with pancreatic tumors undergoing pancreaticoduodenectomy. The aim of this study was to assess the association between preoperative malnutrition and delayed discharge within a ten-day timeframe and potential correlations between preoperative malnutrition and postoperative surgical complications. Methods: A retrospective cohort study was conducted, recruiting a final sample of 79 patients with benign or malignant cephalic pancreatic tumors from 2015 to 2022. The risk of malnutrition was assessed using the Malnutrition Universal Screening Tool, while length of hospital stay and relevant clinical data were extracted from clinical documentation. Results: The preoperative malnutrition risk was high in 21.52% of the sample, moderate in 36.71%, and low in 41.77%. Body mass index (BMI) (*p* = 0.007) and postoperative surgical complications (*p* < 0.001) were significantly correlated with delayed discharge. No statistically significant differences were found between levels of malnutrition risk and delayed discharge (*p* = 0.122), or postoperative surgical complications (*p* = 0.874). Conclusions: Postoperative complications and BMI emerge as significant risk factors. The limited sample size may have compromised the collection of homogeneous and significant data. Future studies should evaluate the implementation of personalized nutritional screening tools, nutritional assessment plans, and the involvement of specialized health professionals.

## 1. Introduction

Pancreatic tumors rank thirteenth in global incidence and seventh in mortality for both sexes and all age groups, with 495,773 new cases annually and 466,003 deaths. The countries with the highest incidence and mortality rates are Asia, Europe, North America, Latin American countries, and Africa [1]. Risk factors for pancreatic cancer include cigarette smoking, alcohol consumption, obesity, diabetes (especially if newly onset), chronic pancreatitis, familial predisposition, and hereditary syndromes like atypical melanoma and Peutz–Jeghers syndrome. Additionally, genetic factors play a crucial role in pancreatic cancer formation, although the mechanisms are not fully understood [2].

Malnutrition is defined as a condition resulting from excess or reduced nutrient intake leading to altered body composition and compromised physical and mental function [3,4]. A more specific concept is disease-related malnutrition (DRM), which develops due to the presence of a disease or related inflammatory state [4]. Individuals with neoplastic diseases often experience a condition called “cancer cachexia”, a chronic multifactorial syndrome characterized by weight loss, muscle mass loss, increased inflammatory and catabolic state, reduced food intake, and refractoriness to conventional nutritional support, affecting about 50% of individuals with neoplastic disease [5,6,7]. This organic state is a continuum characterized by a pre-cachectic phase, cachexia, and refractory cachexia, influenced by cancer type and stage, presence of systemic inflammation, and unresponsiveness to anti-tumor therapy [7]. Neoplastic cachexia affects individuals with pancreatic neoplasms, with approximately 85% experiencing weight loss [5].

Pancreatic neoplasms lead to a systemic inflammatory state capable of modifying and overturning individual muscle composition, damaging pancreatic β cells with consequent altered insulin secretion, and reducing appetite by acting on the hypothalamus [8]. Exocrine pancreatic insufficiency (EPI) is another consequence of pancreatic neoplasms, defined as a multifactorial process due to pancreatic duct obstruction, gland fibrosis, and exocrine tissue loss. This complication results in the reduced excretion of digestive enzymes (amylase, lipase, nucleases, proteases, trypsin, chymotrypsin, elastase, and carboxypeptidase), leading to reduced nutrient absorption [8,9]. Post-surgery, exocrine function further decreases, requiring enzyme supplementation at specific doses during meals. From a mechanical standpoint, there is an extrinsic compression of the stomach, abdominal esophagus, and first duodenal tract, influencing appetite and subsequent weight loss [10]. Preoperative malnutrition is one of the causes of complications in the postoperative period [11,12], and length of stay (LoS) is among the outcomes influenced by malnutrition conditions. Perioperative care has been supported since 2012 by Enhanced Recovery After Surgery (ERAS) guidelines to reduce postoperative complications, shorten hospital stays, and lower healthcare costs [13]. Despite the complexity of this intervention, ERAS guidelines have proven effective in managing patients undergoing this surgical procedure [14].

A recent retrospective observational study [15], defining a LoS of 7 days, described postoperative complications such as pancreatic fistula and compromised intestinal function that prolong hospitalization. It also described how intensive preoperative rehabilitation, including nutritional supplementation, can reduce the previously mentioned complications related to intestinal function. In the context of this study, the LoS for this type of intervention, according to the clinical department protocol, is 8 days. However, since the duration of hospitalization after this surgical intervention does not always reflect the expected duration according to the study context’s care protocol, this research aimed to investigate whether preoperative malnutrition may be correlated with hospitalization beyond the temporal target and with other care outcomes.

### Study Objective

The primary aim of the study is to examine a relationship between patients at risk of malnutrition undergoing pancreaticoduodenectomy (PD) and delayed discharge compared to the temporal parameter established by the postoperative management protocol of the study context. Secondarily, the association between preoperative malnutrition and postoperative surgical complications and risk factors for delayed discharge beyond the target stay was investigated.

## 2. Materials and Methods

### 2.1. Study Design

The study was conducted at the General Surgery Unit, specializing in hepatobiliary-pancreatic surgeries, at Santa Chiara Hospital in Trento, Italy. It employed a retrospective monocentric cohort study design, including patients undergoing pancreaticoduodenectomy (PD) from January 2015 to December 2022. The sample size was determined by convenience and comprised 79 subjects undergoing elective surgery for pancreatic head pathologies. The study adhered to the STrengthening the Reporting of OBservational studies in Epidemiology (STROBE) Guidelines [16].

In adherence to the principles outlined in Good Clinical Practice (GCP) guidelines and the Declaration of Helsinki, ethical approval was obtained from the institutional review board (IRB) and Ethical Committee of Azienda Provinciale per i Servizi Sanitari, Provincia Autonoma di Trento, Italy, under authorization number A882, dated 9 May 2023. Subsequently, the principal investigator contacted the patients by phone to assess their willingness to participate and provide necessary data. Prior to accessing the medical records, a meeting was held to explain the study’s purpose, and consent was obtained through the signing of privacy information and participation consent forms. For patients who could not attend an in-person meeting, consent was obtained over the phone and documented accordingly. For deceased patients, presumed consent was considered in accordance with the IRB’s guidance.

### 2.2. Inclusion and Exclusion Criteria

Inclusion criteria: patients scheduled for elective PD for benign and malignant pathologies who underwent nutritional screening preoperatively.

**Figure 1:**
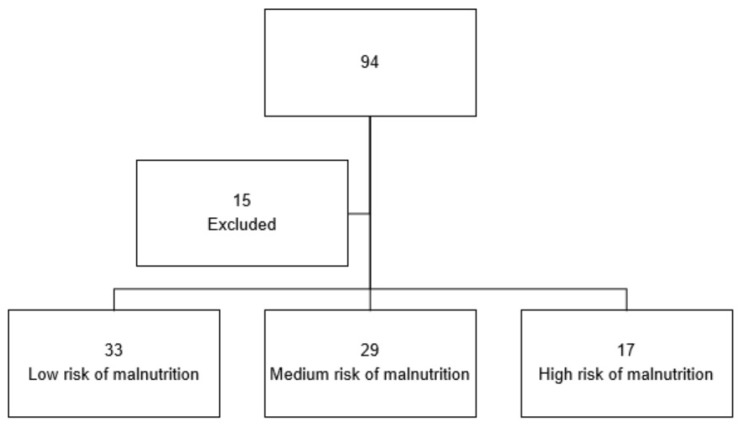
Study sample flow chart. Legend. The figure illustrates the convenience sample selection process. Out of 94 patients undergoing PD from January 2015 to December 2022, 15 patients who did not meet the inclusion criteria were excluded. The remaining patients were categorized based on their malnutrition risk, resulting in 33 low-risk patients, 29 moderate-risk patients, and 17 high-risk patients.

Exclusion criteria: emergency surgery; subjects with peritoneal carcinomatosis found during surgical exploration; patients requiring prolonged hospitalization or deceased due to reasons unrelated to surgery. Patients who could not be contacted to provide research information and consent for participation were also excluded (Figure 1).

### 2.3. Data Collection

Data were collected by reviewing the paper and/or electronic medical records of patients undergoing PD. All clinical data analyzed were collected and organized into an anonymized database protected by a password. The data were anonymously shared with the statistician and research team members. Data collection took place between June 2023 and September 2023.

### 2.4. Study Procedure

**Figure 2:**
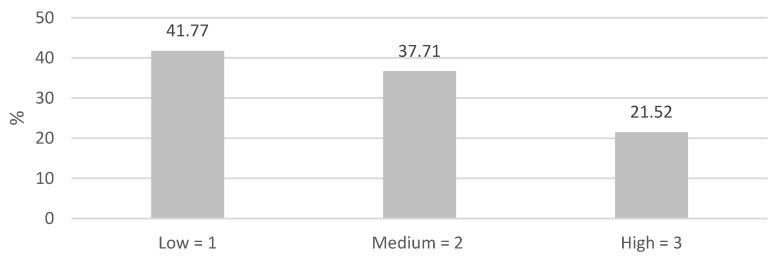
Sample percentage in the three malnutrition risk categories. Legend. The figure represents the frequency of malnutrition risk categorized into three groups according to the Likert scale: low malnutrition risk = 1; moderate malnutrition risk = 2; high malnutrition risk = 3.

In the first phase, preoperative malnutrition risk and length of hospital stay for each analyzed patient were collected. In our hospital setting, the theoretical hospital stay indicated by the clinical management protocol for this type of procedure, adopting an ERAS-based management approach, was 8 days. For the study, a tolerance of +2 days was chosen; hence, the total was 10 days, compared to the previously indicated target to accommodate possible delayed discharges due to logistical issues in returning home, weekends, or waiting for clinical tests for discharge. Sometimes, hospitalization was prolonged due to other factors, such as postoperative complications. Malnutrition risk was systematically assessed in each patient preoperatively using the Malnutrition Universal Screening Tool (MUST) [17], a nutritional screening tool used in the study context. It involves recording body mass index, weight loss in the last 3–6 months, and the presence of acute illness affecting weight loss. Assessments were conducted in the preoperative clinic or during hospitalization. Subsequently, patients were categorized based on the results, and a Likert scale score was assigned to each risk category: low = 1, moderate = 2, high = 3 (Figure 2). Based on the screening results, a specific nutritional supplementation pathway was initiated or not, in anticipation of surgery, following the principles of the ERAS protocol adopted in the study context since 2016. Specifically, upon pre-admission, each patient was educated by an experienced nurse in general surgery on the standard preoperative nutritional preparation, consisting of carbohydrate loading the day before and on the morning of surgery (containing maltodextrin and sucrose without fibers, fats, and proteins; 800 mL, 1672 Kcal, the day before the surgery and 400 mL, 836 Kcal, the morning of the surgery) and immunonutrition (containing Arginine, Omega 3, RiboNucleic Acid, and soluble fiber, gluten-free and containing clinically irrelevant amounts of lactose; 237 mL, 341 Kcal, per brick) 5–7 days before surgery: 1 brick a day if the risk of malnutrition was low, 2 bricks a day if the risk was medium. In the case of an identified high malnutrition risk during hospitalization (MUST score ≥ 2), a personalized nutritional supplementation pathway could be initiated as indicated by the clinical nutrition team.

In the second phase, the search for postoperative complications and their correlation with the three malnutrition risk categories was conducted. In addition to analyzing general postoperative complications, their evaluation was based on prevalence expressed as <3 or ≥3 according to the Clavien–Dindo scale, always in relation to the three malnutrition risk categories. The Clavien–Dindo scale is a tool devised by a multidisciplinary team to create a consensus on the description and classification of postoperative complications [18]. Lastly, potential factors increasing the length of hospital stay were evaluated by analyzing variables such as age (years), gender (male/female), preoperative hematological tests (hemoglobin (g/dL), leukocytes (4 × 10^9^/L), albumin (g/L), total proteins (g/L), carcinoembryonic antigen—CEA (ng/mL), carbohydrate antigen 19.9—CA19.9 (U/mL), C-reactive protein—CRP (mg/L), index normalized ratio—INR), postoperative surgical complications (classified according to the description of postoperative course in discharge letter and Clavien–Dindo scale), preoperative nutritional pathway based on whether no integrative support was provided (for patients treated before 2016 and those operated on during the COVID-19 period), those who followed a standard pathway (carbohydrates loading and immunonutrition), and those who followed a personalized pathway (personalized nutritional treatment) according to the clinical nutrition team’s indications.

### 2.5. Statistical Analysis

The data were presented as numbers and percentages for qualitative variables, mean and standard deviation for approximately normally distributed continuous variables, or median and range for non-normally distributed variables. Gaussian distribution adherence was verified using the Shapiro–Wilk test.

The MUST score was presented using the variable “risk of malnutrition” and was also visualized using a column chart. The MUST scale was completed at the first preoperative meeting by the preoperative nurse, and no further evaluations were conducted after the initial nutritional status assessment or any supplementation period. The length of hospital stay was also considered as a dichotomized variable using a cutoff point of 10 days, with the tenth day as the target hospitalization achieved. The association between nutritional status and hospital stay duration +/− 10 days was assessed using the chi-squared test (χ^2^), while the association with hospital stay duration expressed in days was assessed using the Kruskal–Wallis test when malnutrition risk was considered low, moderate, or high, or with the Mann–Whitney test when the risk was considered low or moderate–high. The association with other categorical variables was evaluated using the chi-squared test (χ^2^). The relative risk of discharge after the target was calculated for each malnutrition risk category, presenting the relative risk with its 95% confidence interval (95% CI) for each category. The prevalence of complications was graphically represented in a histogram, where each column shows the percentage of complications. The association between hospital stay duration beyond 10 days and potential risk factors was explored using logistic regression analysis, and the results were presented as odds ratios (ORs) with their 95% confidence intervals. All analyses were performed using Stata version m17. The significance level was set at 0.05.

## 3. Results

### 3.1. Frequency of Malnutrition Risk and Length of Stay

In the total sample of 79 patients, 33 (41.77%) had a low malnutrition risk, 29 (36.70%) had a moderate malnutrition risk, and 17 (21.51%) had a high malnutrition risk (Figure 2).

Users with a length of stay (LoS) ≤ 10 days totaled 18, while those with a LoS > 10 days totaled 61 (Table 1). Across the three nutritional risk categories, a higher number of participants had a LoS > 10 days compared to those with LoS ≤ 10 days: low risk, 29 participants (47.54%) vs. 4 (22.22%); moderate risk, 21 (34.43%) vs. 8 (44.44%); high risk, 11 (18.03%) vs. 6 (33.33%). However, the relative risk of a LoS beyond the target period in the three nutritional risk classes was not statistically significant (*p* = 0.122) (Table 1).

### 3.2. Postoperative Complications and Risk of Malnutrition

**Figure 3:**
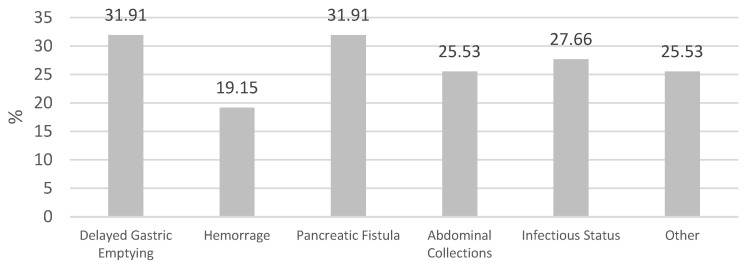
Postoperative surgical complications. Legend. Postoperative surgical complications are represented as a percentage.

The average LoS for each malnutrition risk class was 19 days (range: 7–70 days) for low-risk users, 17 days (range: 7–75 days) for moderate-risk users, and 17 days (range: 7–38 days) for high-risk users. There was no statistically significant difference between the nutritional risk classes and the average LoS (*p* = 0.359). The prevalence of postoperative complications in the study sample is presented in Figure 3.

Specifically, 15 users (31.91%) experienced delayed gastric emptying (DGE); 9 users (19.15%) had postoperative bleeding; 15 users (31.91%) developed pancreatic fistula; 12 users (25.53%) had abdominal collections; 13 users (27.66%) presented with infections; and 12 users (25.53%) experienced other complications (postoperative diabetes, pancreatitis, functional and organic decline, acute renal and respiratory failure, postoperative ascites, or acute myocardial infarction). Overall, there were no significant differences between the nutritional risk classes and postoperative complications (*p* = 0.874) (Table 2).

Users with postoperative surgical complications classified as Clavien–Dindo < 3 totaled twenty-two (eight low-risk, ten moderate-risk, and four high-risk); those with a complication ≥ 3 totaled twenty-five (twelve low-risk, eight moderate-risk, and five high-risk). However, no statistically significant differences were found between the types of postoperative surgical complications and the degree of nutritional risk (Table 3).

### 3.3. Risk Factors and Length of Stay

Regarding risk factors for a LoS ≤ 10 days or >10 days and other collected independent variables, there were no statistically significant correlations for demographic data such as age (*p* = 0.101) and gender (*p* = 0.422). As an anthropometric measure, BMI (body mass index) showed a statistically significantly correlation with the LoS in the two periods (*p* = 0.007). While MUST did not show significant associations with the risk of LoS in the two time categories, high malnutrition risk approached a statistically significant correlation (low, null; moderate, *p* = 0.133; high, *p* = 0.062). There were no statistically significant relationships between users who did not follow a nutritional pathway (*p* = null), those who followed a standard nutritional preparation according to ERAS guidelines (*p* = 0.654), and those who followed a personalized nutritional support pathway supported by clinical nutritionists and dieticians (*p* = 0.353). Additionally, no statistically significant correlations were observed between various laboratory analyses collected and the duration of LoS in the two time categories; only hemoglobin approached the threshold of statistical significance (*p* = 0.052).

Total surgical complications were significantly correlated with a LoS > 10 days (*p* < 0.001); in fact, users who had a LoS in the first time category totaled 1, while those who had a LoS in the second category totaled 46. However, for individual complications compared with the LoS defined by the study, no statistically significant correlations emerged (DGE *p* = 0.131; bleeding, not measurable; fistula, not measurable; abdominal collections *p* = 0.186; infection, not measurable; other complications including post-operative diabetes, pancreatitis, dyslipidemia, Wernicke’s encephalopathy, acute respiratory failure, acute renal failure, post-operative ascites, *p* = 0.223) (Table 4).

## 4. Discussion

The phenomenon of malnutrition within the study context appears to show a uniform distribution across the three risk classes. However, upon further segmentation into low-risk and medium-to-high-risk groups, it becomes evident that the medium-to-high risk of malnutrition, which requires different treatment from simple reevaluation according to the MUST scale indications, represents 58.23% of the sample, thus constituting more than half. The analysis of study findings suggests that preoperative malnutrition risk is not directly correlated to a prolonged postoperative length of stay. Instead, BMI represents a risk factor significantly associated with discharge beyond the temporal threshold defined by the study.

### 4.1. The Body Mass Index as a Risk Factor of Increase in the Length of Stay

BMI serves a measurement index of ideal weight, categorizing individuals as overweight if their measurement is between 25 and 30 kg/m^2^, while above 30 kg/m^2^, obesity is considered [19]. A better assessment of the body is represented by the measurement of body composition. There is a positive correlation between body composition and the genesis of pancreatic cancer, mainly due to the systemic inflammatory response mediated by adipocytes, which influences carcinogenesis [20]. Determining body composition is achievable through various techniques, but BMI, although its measurement is simple, immediate, and low-cost, is not able to detect it. There are many ways to measure body composition, including the use of computed tomography (CT). This latter method allows for the study of body composition by analyzing the differential radiation absorption: the muscle mass is assessed by studying the cross-sectional area of muscles at the level of the third lumbar vertebra, while adipose tissue is evaluated at the same level of section [21,22]. Recent analyses have positively correlated body composition with postoperative complications after duodenocephalopancreasectomy, particularly studying the relationship between visceral fat and muscle mass for their onset, and demonstrating how subjects with an altered ratio between these two variables are at greater risk given their influence on the consistency of the organ itself and the risk of entero-pancreatic anastomotic dehiscence [23,24].

### 4.2. The Postoperative Complications as a Risk Factor of Increase in the Length of Stay

Postoperative complications, recognized for this type of surgical intervention, seem to be another factor more determinant in this study for a delayed discharge compared to the temporal target. Although there is little evidence in the literature regarding the impact of preoperative malnutrition on postoperative outcomes, some research positively correlates the effect of this phenomenon with morbidities and postoperative complications [25,26,27]. Previous studies associated postoperative surgical complications with different factors. For example, pancreatic fistula is correlated with a BMI > 25 kg/m^2^, soft pancreatic consistency, duct of Wirsung diameter < 3 mm, intraoperative transfusion requirements, and male gender [28,29]. Delayed gastric emptying complication is correlated with age, soft pancreatic consistency, preoperative biliary drainage, development of postoperative pancreatic fistula (POPF), presence of abdominal collections, and blood loss. Although statistically significant correlations between length of stay and postoperative complications did not emerge from this study, it is interesting to observe where the highest number of users with surgical complications, according to the Clavien–Dindo scale ≥ 3, is for those at low nutritional risk and decreases for medium and high risk; an inverse trend is present for users with complications < 3, according to the Clavien–Dindo classification. This could be due to the fact that users undergoing this surgical procedure who are more “fit” organically, characterized by a reduced history of pancreatic inflammation, have a softer consistency of this organ and, therefore, are more subject to complications, as indicated by the previous observations.

### 4.3. Method for Assessing the Risk of Malnutrition

During the data collection phase, it emerged that, following initial nutritional screening through the MUST scale, the regular re-evaluation of nutritional status did not occur, nor was an analysis of adherence to personalized indications provided in case of activation of the clinical nutrition team. This lack could be caused by the severity of the disease requiring early intervention to avoid disease progression and by a reduced presence of professionals employed to monitor and manage the preoperative period and therefore the related nutritional aspect. In fact, out of the total sample, it was possible to detect a post-integration weight in 25 users, and of these, 17 had been prescribed a personalized nutritional supplement. In addition, a systematic biomolecular re-evaluation was conducted for a limited number of users to assess the effectiveness of the treatments. Multi-disciplinarity is one of the cornerstones on which user assistance in the perioperative period is based. Various professional figures take part in the decisions, including surgeons, oncologists, anesthesiologists, dietitians, nutritionists, and nurses. The evaluation of the person also includes screening of nutritional status and subsequent activation of dedicated correction pathways/interventions if necessary. Often, this evaluation is entrusted to the pre-hospital nurse, who does not possess advanced skills in or knowledge of nutritional and perioperative surgical care. While the role of specialized nurses in nutritional support is not clearly defined in the literature, some data suggest a reduction in hospital stays among patients receiving assistance from such specialized healthcare professionals [30]. Another contributing factor to the deficiency observed is the absence of a standardized pathway to guide professionals in managing patients regarding nutritional aspects.

### 4.4. The Role of the Preoperative Nutritional Supplementation

ONSs (oral nutritional supplements) are sterile liquid, semi-solid or powder products capable of providing a greater intake of macro and micronutrients [31]. The fields of use are numerous, including intake in the pre-operative period by users who are candidates for surgical therapy. The use of these products, such as preoperative carbohydrate loading, is also indicated for users who are candidates for elective surgery who do not present a nutritional risk [32]. The use of these integrations in the pre-operative period has highlighted how there is an improvement in organic recovery in the post-operative period which could favor an early restoration of functional outcomes that allow the user to return to normal conditions as soon as possible to their daily life activities prior to the operation or allow early adaptation to the conditions resulting from the surgical treatment [33]. A recent systematic review of the literature has demonstrated how nutritional prehabilitation in users undergoing head and neck surgery, including ONS-based treatments, has demonstrated an improvement in postoperative outcomes and the user’s general state of well-being [34]. The ERAS guidelines recommend the use of these products in the preoperative period. Even for pancreatic surgery, the assumption of carbohydrate loading in the pre-operative period is strongly recommended, while the strength of the recommendation remains low regarding products dedicated to immunonutrition [35]. Recent evidence has demonstrated how the use of ONS in the preoperative period, to support individuals with a high nutritional risk, could lead to a benefit in reducing the prevalence of clinically relevant postoperative pancreatic fistula [36].

### 4.5. Limitations

A limitation of this study is represented by the intrinsic characteristic of the study, namely being retrospective; this involves the collection of data that, in the case of non-standardized pathways and evaluation methods, may be different to the extent of being uncollectible. Patients treated between 2015 and 2016, before the implementation of ERAS concepts in perioperative care, might not have undergone the same assessments as those in subsequent years. Furthermore, in this study, users who underwent duodenocephalopancreatectomy in the case of benign and therefore non-systemically aggressive pathology such as malignant neoplasms were included for reasons of sample size. Another limitation is represented by the heterogeneity of the timing through which nutritional screening was performed and the time elapsed before surgery, due to the timing for pre-admission scheduling and subsequent evaluations. Another limit is that it could be represented by a first nutritional evaluation made by an experienced nurse in general surgery and not from a nutritionist. The personalized nutritional treatment is another limit of our study because the heterogeneity of this treatment and the retrospective design of the study did not let us to collect it in a standardized way; despite this, our center, for those with a high risk of malnutrition, we prefer enteral nutrition using personalized ONS, which is different from the ones used in standard care; if it is needed, parenteral nutrition could be added to the supplementation. In addition, it is limiting to associate the influence of preoperative malnutrition alone on overall length of stay, since, given the significant surgical stress related to the intervention, recovery, and the difficulty of regular postoperative feeding, postoperative malnutrition states may have appeared that have influenced the user’s pathway. In future studies, it would be beneficial to consider the presence of this phenomenon, along with other variables, even in the postoperative period, so as to be able to distinguish in chronological order which may have been the most influential malnutrition condition on length of stay. Moreover, this study design offered a limited sample size, which did not always allow for obtaining user subgroups in the considered variables that would allow powerful and significant analyses. Future studies could focus on studying this phenomenon with prospective, multicenter designs with a preliminary calculation of statistical power for the definition of the necessary sample, in order to obtain an adequate sample size for generalized results.

## 5. Conclusions

In our study, preoperative malnutrition did not demonstrate a direct impact on the length of stay beyond the expected temporal target for this type of surgical intervention. Perioperative work protocols for this surgical intervention could consider the use of specialized multi-disciplinary health professionals for pathway management. The creation of personalized nutritional assessment tools should be considered. Future studies could focus on malnutrition as a perioperative phenomenon and assess its potential influence at various stages of the operative pathway.

## Figures and Tables

**Table 1:** Relative risk between LoS and nutritional risk class.

	LoS ≤ 10 Days	LoS > 10 Days	RR (95%CI)
N	18	61	
Risk			*p* = 0.122
Low	4 (22.22%)	29 (47.54%)	1 (ref)
Medium	8 (44.44%)	21 (34.43%)	0.82 (0.64–1.07)
High	6 (33.33%)	11 (18.03%)	0.74 (0.51–1.07)

Legend. LoS = length of stay; RR = relative risk.

**Table 2:** Average LoS and surgical complications in the three risk classes.

	Low Risk	Medium Risk	High Risk	*p*-Value
N	33	29	17	
LoS (days)	19 (7–70)	17 (7–75)	17 (7–38)	0.359
Complications	20 (60.61%)	18 (62.07%)	9 (52.94%)	0.874
Delayed Gastric Emptying	6 (30.00%)	4 (22.22%)	5 (55.56%)	0.217
Hemorrhage	3 (15.00%)	3 (16.67%)	3 (33.33%)	0.495
Pancreatic Fistula	4 (20.00%)	6 (33.33%)	5 (55.56%)	0.147
Abdominal Collections	6 (30.00%)	4 (22.22%)	2 (22.22%)	0.908
Infectious Status	7 (35.00%)	4 (22.22%)	2 (22.22%)	0.691
Other	4 (20.00%)	6 (33.33%)	2 (22.22%)	0.684

Legend. LoS = length of stay.

**Table 3:** Surgical complications according to Clavien–Dindo classification in the three nutritional risk classes.

	Low Risk	Medium Risk	High Risk	*p*-Value
Clavien–Dindo < 3				
Complications	8	10	4	
Delayed Gastric Emptying	4 (50.00%)	3 (30.00%)	3 (75.00%)	0.288
Hemorrhage	0	0	0	
Pancreatic Fistula	2 (25.00%)	4 (40.00%)	2 (50.00%)	0.727
Abdominal Collections	0	2 (20.00%)	0	0.654
Infectious Status	1 (12.50%)	3 (30.00%)	0	0.478
Other	0	3 (30.00%)	1 (25.00%)	0.270
Clavien–Dindo ≥ 3				
Complications	12	8	5	
Delayed Gastric Emptying	2 (16.67%)	1 (12.50%)	2 (40.00%)	0.545
Hemorrhage	3 (25.00%)	3 (37.50%)	3 (60.00%)	0.356
Pancreatic Fistula	2 (16.67%)	2 (25.00%)	3 (60.00%)	0.223
Abdominal Collections	6 (50.00%)	2 (25.00%)	2 (40.00%)	0.589
Infectious Status	6 (50.00%)	1 (12.50%)	2 (40.00%)	0.296
Other	4 (33.33%)	3 (37.50%)	1 (20.00%)	1.000

**Table 4:** Risk factors for the two time categories of postoperative LoS.

	LoS ≤ 10 Days	LoS > 10 Days	OR (95%CI)	*p*-Value
N	18	61		
Demographic Data				
Age (years)	63.1 ± 11.5	67.3 ± 8.5	1.05 (0.99–1.11)	0.101
Gender (M)	9 (50.00%)	37 (60.66%)	1.54 (0.54–4.44)	0.422
Anthropometric Data				
BMI (kg/m^2^)	21.7 ± 3.2	25.4 ± 5.4	1.26 (1.07–1.50)	0.007
MUST				
Low (1)	4 (22.22%)	29 (47.54%)	1	
Medium (2)	8 (44.44%)	21 (34.43%)	0.36 (0.10–1.36)	0.133
High (3)	6 (33.33%)	11 (18.03%)	0.25 (0.06–1.07)	0.062
Nutritional Pathway				
No	4 (22.22%)	12 (20.34%)	1	
Standard	10 (55.56%)	22 (37.29%)	0.73 (0.19–2.85)	0.654
Personalized	4 (22.22%)	25 (42.37%)	2.08 (0.44–9.79)	0.353
Laboratory Analysis				
Hemoglobin (g/dL)	13.2 ± 1.4	12.3 ± 1.7	0.71 (0.51–1.00)	0.052
White Blood Cells (×10^9^/L)	7.04 ± 2.73	7.26 ± 4.33	1.01 (0.88–1.17)	0.841
Albumin (g/L)	34.2 ± 4.3	32.4 ± 5.4	0.93 (0.83–1.05)	0.243
Total Proteins (g/L)	64.9 ± 5.9	65.2 ± 7.1	1.01 (0.92–1.09)	0.907
CEA (ng/L)	3.79 ± 6.822.1 (0–29.5)	2.17 ± 2.011.5 (0.5–10.8)	0.91 (0.77–1.08)	0.267
CA19.9 (U/mL)	190 ± 28080.3 (7.8–1052)	101 ± 17135.3 (1.9–897.3)	0.998 (0.995–1.001)	0.172
CRP (mg/L)	5.04 ± 2.914.86 (0–9.9)	20.08 ± 32.568.7 (0–136)	1.10 (0.94–1.29)	0.228
INR	1.07 ± 0.25	1.07 ± 0.13	1.06 (0.04–27.52)	0.973
Complications				
Complications	1 (5.56%)	46 (75.41%)	52.1 (6.4–425.4)	<0.001
Delayed Gastric Emptying	1 (5.56%)	14 (22.95%)	5.06 (0.62–41.49)	0.131
Hemorrhage	0	9 (14.75%)	NA	
Pancreatic Fistula	0	15 (24.59%)	NA	
Abdominal Collections	1 (5.56%)	12 (19.57%)	4.16 (0.50–34.45)	0.186
Infectious Status	0	13 (21.31%)	NA	
Other	1 (5.56%)	11 (18.03%)	3.74 (0.45–31.15)	0.223

Legend. LoS = length of stay; OR = odds ratio; BMI = body mass index; CEA = carcinoembryonic antigen; CA19.9 = cancer antigen 19.9; CRP = C-reactive protein; INR = international normalized ratio; NA = not available.

## Data Availability

Data are available will be available upon request from the corresponding author due to ethical and legal restrictions.
